# Mass spectrometry analysis of PPIP5K1 interactions and data on cell motility of PPIP5K1-deficient cells

**DOI:** 10.1016/j.dib.2016.03.035

**Published:** 2016-04-02

**Authors:** Gayane Machkalyan, Phan Trieu, Darlaine Pétrin, Terence E. Hébert, Gregory J. Miller

**Affiliations:** a Department of Pharmacology and Therapeutics, McGill University, Montréal, Québec, Canada; b Department of Chemistry, The Catholic University of America, Washington, DC, USA

## Abstract

Inositol pyrophosphates are cellular signals that are created by the actions of inositol kinases and are degraded by highly active inositol phosphatases. The potent actions of these phosphatases suggest these signals must be created near their sites of action. To identify sites where the inositol kinase, PPIP5K1 acts, we performed affinity purification of PPIP5K1 from HEK293 cells and analyzed these samples using mass spectrometry to identify the proteins pesent (10.1016/j.cellsig.2016.02.002) [1]. We further decreased PPIP5K1 levels in HeLa cells and treated these with PPIP5K1 siRNA. We then monitored the motility of these cells in Scratch assays.

**Specifications Table**

**Table:** 

Subject area	*Biology*
More specific subject area	*Cell biology*
Type of data	*Table, Movie files*
How data was acquired	*Mass spectroscopy, Microscope* (Operetta High Content microscope (Perkin Elmer, Woodbridge, ON, Canada))
Data format	*Filtered*
Experimental factors	*Affinity-tagged PPIP5K1 was purified from cultured HEK293 cells. HeLa cells were treated with PPIP5K1 siRNA*
Experimental features	*PPIP5K1 and co-purified proteins were analyzed and identified using mass spectrometry. HeLa cells treated with PPIP5K siRNA were subjected to a Scratch test and their motility was monitored by video*
Data source location	*Montreal, Canada*
Data accessibility	*Data is with this article*

**Value of the data**

• Reports cellular interactions with PPIP5K1
• The data contributes to the interaction network of the cell and cell signaling
• The data may further stimulate investigations of inositol signaling in cell motility

## 1 Data

The data in this article is the analysis of proteomic data of the interactions between PPIP5K1 and other cellular proteins [1]. Also included here are videos of the motility of HeLa cells with normal and decreased levels of PPIP5K1.

## 2 Experimental design, materials and methods

### 2.1 Preparation of cell lysates

HEK 293F cell lines stably expressing full length PPIP5K1 WT and PPIP5K1 ΔC constructs were grown on 6 well plates until cells reached 80% confluency. Plates were then placed on ice, washed twice with ice-cold phosphate-buffered saline (PBS) (137 mM NaCl, 2.7 mM KCl, 10 mM Na_2_HPO_4_, 2 mM KH_2_PO_4_), and resuspended in 300 μl lysis buffer (50 mM Tris–HCl, pH 7.4, 150 mM NaCl, 1% NP-40, 2 mM EDTA, 2 mM EGTA, 1 mM DTT, 2 mM NaF, 2 mM NaVO_3_, 1 mM PMSF) and a protein inhibitor cocktail (Roche Diagnostics, Indianapolis, Indiana, USA). Cells were lysed for 1 h with gentle rotation at 4 °C followed by sonication at low power. Lysates were cleared by centrifugation at 3000*g* for 10 min. Protein concentrations were measured using the Bradford protein assay (Bio-Rad, Mississauga, Ontario, Canada) using BSA as standard and 25 μg of protein was used for western blot analysis. HeLa cells transiently transfected with 25 nM siRNAs were grown on 6 well plate for 48 h. Plates were placed on ice and cells were washed twice with ice-cold PBS and resuspended in 300 μl of 2x Laemmli buffer (125 mM Tris–HCl pH 6.8, 2% SDS, 20% glycerol, 4% β-mercaptoethanol) and heated to 65 °C for 15 min, and 40 μl of total lysates were analyzed by western blotting.

### 2.2 Affinity purification

Tagged PPIP5K1 WT and PPIP5K1 ΔC constructs contained a hemagglutinin (HA) tag, streptavidin binding domain (SBD), TEV (tobacco etch virus) protease cleavage site, and a calmodulin-binding domain (CBD) at their N-termini. The purification protocol was adapted from previously published protocol from our group [2]. T175 flasks were placed on ice and cells were washed twice with ice-cold PBS. Cells were detached with PBS-EDTA (PBS with 5 mM EDTA) and pelleted by centrifugation at 400*g* for 5 min. Cells were resuspended in lysis buffer (10% glycerol, 0.1% NP-40, 50 mM, HEPES pH 8.0, 150 mM NaCl, 2 mM EGTA, 1 mM EDTA, 2 mM DTT, 1 mM NaF, 2 mM NaVO_3_) with protease inhibitor cocktail (Complete EDTA free tablet from Roche). Cells were incubated at 4 °C with a constant rotation for 30 min, then frozen at −80 °C, thawed on ice, and centrifuged at 10,000*g* for 10 min. Cleared lysates containing 5 mg of total protein were incubated with 150 μl of streptavidin beads for 16 h. Streptavidin beads were washed 3 times with 1 ml of wash buffer (50 mM HEPES, pH 8.0, 150 mM NaCl, 0.1% NP40, 2 mM EGTA, 1 mM EDTA, 2 mM DTT). Proteins bound to streptavidin beads were eluted using 200 μl of elution buffer (50 mM HEPES, pH 8.0, 150 mM NaCl, 1 mM EDTA, 2 mM DTT, 20 mM biotin). A small fraction of the eluate (25 μl) was retained to analyze efficiency of the purification by western blot analysis.

### 2.3 Sample preparation and mass spectrometry

Detergent was removed from samples by TCA precipitation [3]. Proteins were solubilized, digested by trypsin and samples were prepared by IRCM Proteomics platform as previously described [4]. LC–MS/MS mass spectrometry and protein identification was performed by IRCM Protein platform was performed as described previously [4].

### 2.4 Protein selection

Proteins present in PPIP5K1 WT and PPIP5K1 ΔC eluates were filtered in a two-step screening process. In the first step, we calculated a fold change (FC) of ratio of average normalized spectral counts of identified proteins in negative control (mock transfected HEK 293F cells) versus bait (PPIP5K1 WT and PPIP5K1 ΔC) purifications as described previously [5]. Proteins that were abundant (i.e., FC 40 mg/well value over 1) in the negative control were eliminated. The remaining list of proteins was compared to common contaminants found in more than 15% of experiments in proteomic experiments using HEK 293 cells based on the peer-annotated CRAPome database [6].

### 2.5 Scratch assays

HeLa cells (4×10^5^ cells per well in 6 well plate) were transiently transfected with siRNAs for 24 h, then trypsinized and plated (2×10^5^ cells/well) on fibronectin coated 12-well plates (Corning, Corning, NY, USA). Lyophilized fibronectin (Roche Applied Science, Laval, Quebec, Canada) was resuspended in sterile water and applied to 12 well plates (40 ▯g/well) for 30 min at 37 C and washed once with sterile PBS prior adding media and cells. HeLa cells were grown to confluency and scratches were made to a monolayer with micropipette tip. Cells were washed with sterile PBS and fresh DMEM media with 10% FBS was added to each well. Cells were imaged in an Operetta High Content microscope (Perkin Elmer, Woodbridge, ON, Canada) and images were taken at 10x magnification in controlled condition (37 °C and 5% CO_2_) for 18 h. Wound closing and kinetic properties of migrating cells were analyzed with Columbus Image Data Storage and Analysis software (Perkin Elmer, Woodbridge, ON, Canada).

## Appendix A Supplementary material

Supplementary material

Supplementary material **Movie M1***Scratch assay of HeLa cells treated with control siRNA*. HeLa cells transfected with 25 nM non-targeting siRNAs (siCtrl) and pEGFP were grown to a monolayer and scratches were made with a micropipette tip. Wound closing was monitored for 15 h post-scratch as cells were placed in live-cell chamber under controlled conditions (37 °C and 5% CO_2_). Real-time images were taken with an Operetta High Content Microscope (Perkin Elmer, Woodbridge, Ontario).

Supplementary material **Movie M2***Scratch assay of HeLa cells treated with siPPIP5K1.* HeLa cells transfected with siRNA targeting PPIP5K1and EGFP empty vector were grown to a monolayer and scratches were made with a micropipette tip. Wound closing was monitored for 15 hours post scratch as cells were placed in live-cell chamber under controlled conditions (37 °C and 5% CO_2_). Real-time images were taken with an Operetta High Content Microscope (Perkin Elmer Woodbridge, ON, Canada).

Supplementary material **Movie M3***Scratch assay of HeLa cells treated siPPIP5K1 and co-transfected with PPIP5K1.* HeLa cells transfected with siRNA targeting PPIP5K1 and PPIP5K1 WT vector were grown to a monolayer and scratches were made with a micropipette tip. Wound closing was monitored for 15 h post scratch as cells were placed in live-cell chamber under controlled conditions (37°C and 5% CO_2_). Real-time images were taken with an Operetta High Content Microscope (Perkin Elmer Woodbridge, ON, Canada).
