# Supplementary material for: Mass spectrometry analysis of PPIP5K1 interactions and data on cell motility of PPIP5K1-deficient cells
Source: Data Brief. 2016 Apr 2;7:1443–6. doi: 10.1016/j.dib.2016.03.035 (PMC5063796; doi:10.1016/j.dib.2016.03.035)
Supplement: Supplementary file 1 — Supplementary material [file mmc1.zip › Data in Brief_Proteomics/CS_exocyst_supplementary-2-2.docx]

**Supplementary material**

**Table S1**: *Proteins identified to interact with PPIP5K1.* Shown are proteins identified to interact with PPIP5K1WT and PPIP5K1ΔC proteins using affinity purification and LC-MS analysis (n=3). Proteins are listed in alphabetical order according to their gene name. Identified proteins are listed according to Uniprot accession number as of UniProtKB-SwissProt database accession number (December 2014). The three columns “Biological process”, “Molecular function” and “Cellular component” are Gene Ontology (GO) terms as annotated in GO database and retrieved from Gene Ontology Consortium database. Cellular component abbreviations: PM-plasma membrane, C-cytosol, CP-cytoplasm, ER-endoplasmic reticulum, M-mitochondria, CK-cytoskeleton, GA-Golgi apparatus, GM-Golgi membrane, IM-integral to membrane, N-nucleus, NP-nucleoplasm, CJ-cell junction, AP-apical junction; MT-microtubule, CS-centrosome; MB-midbody; ECR-extracelular region; EXC-exocyst; D-desmosome; FA- focal adhesion ; NP-nuclear pore; LF- laminal filament

| **UniProt accession number** | **Gene ID** | **Identified Proteins** | **WT PPIPK1**  **N. of unique peptides** | **WT PPIPK1**  **% Protein coverage** | **ΔC PPIPK1**  **N. of unique peptides** | **ΔC PPIPK1**  **% Protein coverage** | **Biological Process** | **Molecular function** | **Cellular Component** |
| --- | --- | --- | --- | --- | --- | --- | --- | --- | --- |
| [P55265](http://www.proteinmodelportal.org/query/uniprot/P55265) | ADAR | Adenosine deaminase, RNA-specific, isoform CRA_b | 0 | 0 | 4 | 3 | RNA processing | Double-stranded RNA adenosine deaminase activity | N |
| AAH42998 | ADD1 | Alpha-adducin | 4 | 8 | 0 | 0 | Actin cytoskeleton organization | Actin binding |  |
| [Q12802](http://www.uniprot.org/uniprot/Q12802) | AKAP13 | Guanine nucleotide exchange factor Lbc | 4 | 1.1 | 0 | 0 | Apoptotic signaling pathway  Neutrophin TRK receptor signaling pathway  Regulation of Rho protein signal transduction  Regulation of small GTPase mediated signal transduction | cAMP-dependent protein kinase activity  Phospholopid binding  Rho guanyl nucleotide exchange factor activity  Signal transducer activity | CP; C; M; N |
| Q92974 | ARHGEF2 | Rho/Rac guanine nucleotide exchange factor (GEF) 2, isoform CRA_a | 10 | 5.9 | 0 | 0 | Actin filament organization  Apoptotic signaling pathway  Cell division  Cellular response to hyperosmotic stress  Small GTPase mediated signal transduction | Microtubule binding  Phospholipid binding  RacGTPase binding  Rho guanyl-nucleotide exchange factor activity | C, GA, PM |
| P54252 | ATXN3 | Ataxin 3 variant | 0 | 0 | 1 | 2.4 | Actin cytoskeleton organization; protein de-ubiquitination | Ubiquitin-specific protease activity; ATPase activity | CP;N |
| [Q86U38](http://www.uniprot.org/uniprot/Q86U38) | C14orf21 | Chromosome 14 open reading frame 21, isoform CRA_a | 2 | 5.4 | 1 | 2.8 |  | RNA-binding | N |
| Q9HC52 | CBX8 | Chromobox protein homolog 3 | 1 | 7.1 | 2 | 15 | Histone ubiquitination; negative regulation of transcription | Ubiquitin-protein transferase activity; methylated histone binding | N |
| Q5T0F9 | Cc2d1b | Coiled-coil and C2 domain containing 1B, isoform CRA_b | 1 | 2.7 | 0 | 0 | Transcription regulation | RNA polymerase II core promoter proximal region sequence-specific DNA binding | N |
| [B9EGA7](http://www.uniprot.org/uniprot/B9EGA7) | CDC42BPA | CDC42BPA protein | 7 | 5.3 | 0 | 0 | Actin cytoskeleton reorganization  Intracellular signal transduction | ATP binding  Metal ion binding  Phospholipid binding  Protein Serine/Threonine kinase activity  Small GTPase regulator activity |  |
| P55287 | CDH11 | Cadherin 11, type 2, OB-cadherin (osteoblast), isoform CRA_b | 0 | 0 | 1 | 6.6 | Cell adhesion | Calcium ion binding | PM; CP |
| [P99999](http://www.uniprot.org/uniprot/P99999) | CYCS | Cytochrome c | 2 | 13 | 3 | 24 | Apoptotic DNA fragmentation  Apoptotic process | Protein phosphatase type 2A complex  Respiratory chain  Protein binding | C; M, N |
| [O00273](http://www.uniprot.org/uniprot/O00273) | DFFA | DNA fragmentation factor, 45kDa, alpha polypeptide, isoform CRA_a | 2 | 7.5 | 0 | 0 | Apoptotic process  Apoptotic DNA fragmentation  Signal transduction | Deoxyribonuclease activity | CP; C; N |
| [Q5JSL3](http://www.uniprot.org/uniprot/Q5JSL3) | DOCK11 | Dedicator of cytokinesis protein 11 | 4 | 1.4 | 0 | 0 | Blood coagulation  Small GTPase mediated signal transduction | Phospholipid binding  Rho GTPase binding | C |
| Q9C005 | DPY30 | Protein dpy-30 homolog | 1 | 16 | 0 | 0 | Endosomal transport; transcription regulation | Protein homodimerization activity | GA; N |
| Q9H8V3 | ECT2 | Epithelial cell transforming 2 | 8 | 8.9 | 0 | 0 | Cell morphogenesis  Cytokinesis  Intracellular signal transduction  Regulation of Rho protein signal transduction | GTPase activator activity  Phospholipid binding  Rho guanyl-nucleotide exchange factor | CP; N |
| Q9HCM4 | EPB41L5 | KIAA1548 protein | 4 | 4 | 0 | 0 | Actomyosin Structure organization  Apical construction  Cell migration  Cellular response to TGF-beta  Embryo development | Cytoskeletal protein binding  Protein domain specific binding | PM; CK; CP; FM |
| Q9BSJ8 | ESYT1 | Extended synaptotagmin-1 | 5 | 6 | 0 | 0 | Transport; lipid transport | Protein binding | IM |
| Q9NV70 | EXOC1 | Exocyst complex component 1 | 20 | 14 | 0 | 0 | Cellular membrane organization  Cellular protein metabolic process  Exocytosis  Protein transport |  | PM. EXC |
| [Q96KP1](http://www.uniprot.org/uniprot/Q96KP1) | EXOC2 | Exocyst complex component 2 | 42 | 29 | 0 | 0 | RalGTPase binding  Exocytosis  Protein transport | Protein binding | PM |
| O60645 | EXOC3 | Exocyst complex component 3 | 21 | 16 | 0 | 0 | Protein transport  Exocytosis | Protein binding | EXC |
| Q96A65 | EXOC4 | KIAA1699 protein | 44 | 31 | 0 | 0 | Protein transport  Exocytosis  Cellular membrane organization | Protein binding  PDZ domain binding | PM; EXC |
| [O00471](http://www.uniprot.org/uniprot/O00471) | EXOC5 | Exocyst complex component 5 | 21 | 21 | 0 | 0 | Protein transport  Exocytosis  Cellular membrane organization | Protein binding | PM; C |
| [Q8TAG9](http://www.uniprot.org/uniprot/Q8TAG9) | EXOC6 | Exocyst complex component 6, isoform CRA_a | 6 | 6.6 | 0 | 0 | Erythrocyte differentiation  Vesicle docking involved in exocytosis  Protein transport | Protein binding | PM; EXC |
| [Q9Y2D4](http://www.uniprot.org/uniprot/Q9Y2D4) | EXOC6B | Exocyst complex component 6B | 15 | 13 | 0 | 0 | Vesicle docking involved in exocytosis  Protein transport | Protein binding | PM; EXC |
| Q9UPT5 | EXOC7 | Exocyst complex component 7 | 41 | 36 | 0 | 0 | Cellular membrane organization  Cellular protein metabolic process  Exocytosis  Protein transport | Protein binding | PM; C;EXC |
| [Q8IYI6](http://www.uniprot.org/uniprot/Q8IYI6) | EXOC8 | Exocyst complex component 8 | 33 | 25 | 0 | 0 | Vesicle docking involved in exocytosis  Protein transport | Protein binding | PM; EXC, N |
| A0FGR8 | FAM62B | Family with sequence similarity 62 (C2 domain containing) member B, isoform CRA_a | 4 | 5 | 0 | 0 | Lipid transport; transport | Lipid binding  Protein binding | PM |
| [Q9H4G4](http://www.uniprot.org/uniprot/Q9H4G4) | GLIPR2 | Golgi-associated plant pathogenesis-related protein 1 | 3 | 17 | 0 | 0 |  | IP_6_ binding | GM |
| P12081 | HARS | Histidine T-RNA ligase | 2 | 7.6 | 0 | 0 | tRNA aminoacetylation for protein translation | Aminoacyl-tRNA ligase activity  ATP binding | CP |
| P52926 | HMGA2 | High mobility group protein HMGI-C isoform a | 0 | 0 | 2 | 39 | Transcription regulation | DNA binding | N |
| Q6P1M3 | L2GL2 | Lethal giant larvae homolog 2 (Drosophila), isoform CRA_a | 3 | 3.3 | 0 | 0 | Cell cycle  Cell division  Exocytosis  Establishment or maintenance of cell polarity | PDZ domain binding  Protein binding | CP |
| Q14847 | LASP1 | LIM and SH3 protein 1, isoform CRA_a | 1 | 3.1 | 5 | 11 | Ion transport  Cortical actin cytoskeleton | Actin filament binding  SH3/SH2 adaptor activity  Zn ion binding | FA |
| Q6ZN17 | LIN28B | Lin-28 homolog B (C. elegans), isoform | 0 | 0 | 5 | 17 | miRNA catabolic process  pre-miRNA processing  regulation of transcription  RNA 3”-end processing | DNA binding  Protein binding  RNA binding  Zn ion binding | CP; M  N |
| Q6WCQ1 | MPRIP | Myosin phosphatase-Rho interacting protein, | 8 | 5.7 | 0 | 0 |  | Actin binding  Phospholipid binding | CP; CK |
| Q9H1R3 | MYLK2 | Myosin light chain kinase 2, skeletal muscle, isoform CRA_a | 2 | 2.6 | 2 | 2.6 | Cardiac cell differentiation, contraction  Neuromascular synaptic transmission | ATP binding  Camodulin Binding | CP; N |
| O43795 | MYO1B | Unconventional myosin-Ib isoform 1 | 34 | 20 | 0 | 0 | Actin filament binding; post-Golgi mediated vesicle transport | Actin filament binding; phosphatidylinositol 4,5 –biphosphate binding | PM; CP; AF |
| O94832 | MYO1D | Myosin ID, isoform CRA_a | 19 | 25 | 0 | 0 | Early endosome to recycling endosome transport | Actin-dependent ATP activity |  |
| P35658 | NUP214 | Nucleoporin 214kDa, isoform CRA_b | 0 | 0 | 2 | 1.9 | mRNA export from nucleus  protein transport from nucleus, Cell cycle | Nucleocytoplasmic transporter activity | NP |
| P37198 | NUP62 | Nuclear pore glycoprotein p62 | 0 | 0 | 3 | 6.1 | Cell aging  Negative regulation of epidermal growth pathway | PTB domain binding | NP |
| Q99567 | Nup88 | 88kDa nuclear pore complex protein | 0 | 0 | 5 | 6.9 | Cytokine-mediated signaling pathway  Glucose transport  mRNA transport  protein transport | Transporter activity | C, N,NP |
| P78356 | PIP4K2A | Phosphatidylinositol 4-phosphate 5-kinase type-1 | 34 | 34 | 0 | 0 | Cell surface receptor signaling pathway  Phosphatidylionositol biosynthetic process | 1-phosphatidylinositol-4-phosphate-5-kinase activity  1-phosphatidylinositol-5-phosphate-4-kinase activity  ATP-binding  receptorsignalling protein activity | C; ER; N; PM |
| O14986 | PIP4K2B | Phosphatidylinositol 5-phosphate 4-kinase type-2 beta | 4 | 15 | 0 | 0 | Cell surface receptor signaling pathway  Phosphatidylionositol biosynthetic process | 1-phosphatidylinositol-4-phosphate-5-kinase activity  1-phosphatidylinositol-5-phosphate-4-kinase activity  ATP-binding  Receptor signalling protein activity | PM; C; ER; N |
| [O60331](http://www.uniprot.org/uniprot/O60331) | PIP5K1C | Chain A, Human Phosphatidylinositol-4-Phosphate 5-Kinase, Type Ii, Gamma | 16 | 21 | 0 | 0 | Cell adhesion  Chemotaxis  Endocytosis  Exocytosis  Phagocytosis | Phosphatidylinositol phosphate kinase activity  ATP-binding | CJ, CM, C, PM, N |
| Q16513 | PKN2 | Protein kinase N2, isoform CRA_c | 8 | 7.4 | 0 | 0 | Signal transduction  Apoptosis  Cell cycle | Kinase activity | CP |
| Q6P5Z2 | PKN3 | Serine/threonine-protein kinase N3 | 7 | 10 | 0 | 0 | Epithelial cell migration  Protein phosphorylation  Signal transduction | ATP binding  Protein binding  Protein kinase activity | GA; N |
| O95602 | POLR1A | DNA-directed RNA polymerase I subunit RPA12 | 0 | 0 | 1 | 13 | Gene expression | DNA binding, zinc ion binding | N |
| Q6PFW1 | PPIP5K1 | inositolhexakisphosphate and diphosphoinositol-pentakisphosphate kinase 1 isoform 5 | 2 | 1.5 | 188 | 39 | Bait | Bait | Bait |
| Q13131 | PRKAA1 | Protein kinase, AMP-activated, alpha 1 catalytic subunit | 5 | 6.1 | 0 | 0 | Signal transduction  Apoptosis  Cell cycle |  | CP |
| Q05655 | PRKCD | Protein kinase C delta type | 39 | 34 | 0 | 0 | Intracellular signal transduction  Cellular response to glucose starvation  Positive regulation of MAP kinase activity | Ca-independent protein kinase C ativity  Insulin receptor substrate binding  Protein kinase binding  TIR domain binding | PM;CJ; C; M |
| P05129 | PRKCG | Protein kinase C, theta, isoform CRA_a | 5 | 12 | 0 | 0 | Intracellular signal transduction | Intracellular signal transduction | PM; C |
| Q15139 | PRKD1 | Serine/threonine-protein kinase D1 | 5 | 4.3 | 0 | 0 | Intracellular signal transduction | ATP binding  Metal ion binding  Phospholipid binding  Protein kinase C activity | CP |
| Q12923 | PTPN13 | Protein tyrosine phosphatase, non-receptor type 13 (APO-1/CD95 (Fas)-associated phosphatase), isoform CRA_a | 54 | 18 | 0 | 0 | Protein dephosphorylating | Protein binding  Protein tyrosine phosphatase activity | PM; CK; CP; N |
| P10586 | PTPRF | Protein tyrosine phosphatase, receptor type, f polypeptide (PTPRF), interacting protein (liprin), alpha 1 | 5 | 4.6 | 0 | 0 | Peptidyl-tyrosine dephosphorylation  Transmembrane receptor protein tyrosine phosphatase signaling pathway | Heparin binding  Protein tyrosine phosphatase activity  Transmembrane receptor protein tyrosine phosphatase activity | PM |
| Q9ULC3 | RAB23 | Ras-related protein Rab-23 | 4 | 21 | 0 | 0 | Autophagic vacuole assembly  Cellular defense response  GTP catabolic process  Protein transport  Small GTPase mediated signal transduction | GTP binding  GTPase activity | PM, CP |
| Q15286 | RAB35 | Ras-related protein Rab-35 isoform 1 | 4 | 27 | 0 | 0 | Cytokinesis  Endosomal transport  GTP catabolic process  Protein transport  Small GTPase mediated signal transduction | GTP binding  GTPase activity  PI4,5 binding | PM;CP |
| P61006 | RAB8A | Ras-related protein Rab-8A | 6 | 24 | 0 | 0 | Axonogenesis  Cilium assembly  G2/M transition of mitotic cell cycle  GTP catabolic process  Protein transport  Small GTPase mediated signal transduction | GTP binding  GTPase activity  RabGTPase binding | PM; CP |
| P78406 | RAE1 | mRNA-associated protein mrnp41 | 0 | 0 | 5 | 12 | Carbohydrate metabolic process  Cellular response to organic  cytokine-mediated signaling pathway  mitotic cell cycle  trans-membrane process | Microtubule binding  RNA binding | CP; N |
| Q9P0K7 | RAI14 | Ankycorbin | 21 | 14 | 0 | 0 |  |  | C, M, N |
| P11233 | RALA | Ras related protein Ral_A | 6 | 26 | 0 | 0 | Apoptotic process  Cytokinesis  Regulation of exocyst assembly  Ras protein signal transduction | GTPase activity  GTP binding  Protein binding | PM. MB |
| P18754 | RCC1 | Regulator of chromosome condensation isoform a | 0 | 0 | 8 | 15 | Cell division  Chromosome condensation  G1/S transition of mitotic cell cycle  Viral process | Chromatin binding  Histone binding  Ran guanyl-nucleotide exchange factor activity | CP; N |
| **P61586** | RHOA | Transforming protein RHOA | 24 | 48 | 0 | 0 | GTP catabolic process; Rho protein signal transducstion; actin cytoskeleton organization; cell cycle, NFkb import to nucelus | GTP binding, GTPase activity; protein binding, myosin binding | AJ ; CJ, C, CK; PM |
| Q96MS0 | ROBO3 | Roundabout, axon guidance receptor, homolog 3 (Drosophila), isoform CRA_d | 1 | 0.98 | 0 | 0 | Axon guidance; neuron migration | Developmental protein | PM |
| Q9NQG5 | RPRD1B | Regulation of nuclear pre-mRNA domain-containing protein 1B | 1 | 4.6 | 4 | 16 | Positive regulation of cell proliferation  Positive regulation of transcription RNA polymerase II promoter  Regulation of cell cycle process | Protein binding  RNA polymerase II binding | N |
| P31151 | S100A7 | Protein S100-A7 | 0 | 0 | 4 | 23 | Angiogenesis  Immune response positive regulation of ER1/2 cascade | Ca ion binding  Protein binding  RAGE receptor binding  Zn ion binding | FA; CP; ER; N |
| P05109 | S100A8 | Protein S100-A8 | 4 | 24 | 4 | 24 | Activation of cysteine-type-endopeptidase activity  Immune response  Autophagy  Inflammatory response | Arachidonicacid binding  Protein binding  RAGE receptor binding  Zn ion binding  Toll-like receptor binding | PM; CK, C |
| P06702 | S100A9 | Protein S100_A9 | 4 | 25 | 4 | 25 | Activation of cysteine-type-endopeptidase activity involved in apoptosis  Autophagy  Cell-cell signaling  Chemokine production | Antioxidant activity  Arachidonic acid bining  Ca ion binding  RAGE receptor binding  Signal transducer activity  Toll-like receptor 4 binding  Zn ion bining | C; CK; ECR; PM |
| Q6UWP8 | SBSN | Suprabasin isoform 1 precursor | 2 | 3.1 | 4 | 6.1 |  |  | CP; ECR |
| O00560 | SDCBP | Syndecan binding protein (syntenin), isoform CRA_a | 2 | 7.9 | 0 | 0 | Actin cytoskeleton organization | Cytoskeletal adaptor activity | PM; CP;N |
| [Q9P0U3](http://www.uniprot.org/uniprot/Q9P0U3) | SENP1 | Chain B, Senp1 (Mutant) Full Length Sumo1 | 0 | 0 | 4 | 23 | Apoptotic signaling pathway  Negative regulation of proteosomal ubiquitin-dependent catabolic process  Protein sumoylation | Endopeptidase activity  SUMO-specific protease activity | CP  NP; N |
| Q96P63 | SERPINB12 | Serpin B12 | 4 | 14 | 3 | 14 | Regulation of proteolysis | Enzyme binding | CP |
| Q4KMP7 | TBC1D10B | TBC1 domain family member 10B | 9 | 12 | 0 | 0 | Rab GTPase activator activity | RabGTPase activator activity | CP |
| P51784 | USP11 | Ubiquitin specific peptidase 11, isoform CRA_b | 2 | 2.6 | 1 | 1.3 | Protein deubiquitination | Cysteine-type endopeptiodase activity  Protein binding | CP;N |
| Q93008 | USP9X | ubiquitin specific peptidase 9, X-linked, isoform CRA_a | 2 | 0.54 | 1 | 0.54 | BMP signaling pathway  Cell division  Chromosome segregation  Gene expression  Protein deubiquitination  Transcription DNA-dependent | Co-SMAD binding  Cysteine-type endopeptiodase activity  Protein binding  Ubiquitin thiolesterase activity | CP |
| Q96F45 | ZNF503 | zinc finger protein 503, isoform CRA_b | 1 | 2 | 4 | 10 | Transcription regulation | Metal ion binding | CP; N |
| Q9H7S9 | ZNF703 | zinc finger protein 703 | 0 | 0 | 1 | 4.2 | Transcription regulation | Metal ion binding | CP; N |

*Generating the Virtual Protein Network of PPIP5K1*

1. Proteins identified to bind to PPIP5K1 WT and PPIP5K1 ΔC constructs by mass spectrometry were screened to make sure that they were not “frequent flyers”. First their abundance in the PPIP5K1 samples was compared to controls and if they were equal or higher in control, they were eliminated. In the next step, proteins that were more abundant in PPIP5K1 constructs were entered into the web-based CRAPome database and proteins that were identified in more than 15% of Streptavidin agarose purification assays were eliminated as well. This screening process reduced the list of proteins interacting with PPIP5K1 WT to 61 and PPIP5K1 ΔC to 28.
2. We created Excel spreadsheets for each PPIP5K1 construct and proteins interacting with them in a format that would be compatible with Cytoscape software to generate networks. (Supplementary file: CS_PPIP5K1.elsx)
3. We created PPIP5K1 WT construct network by importing Excel spreadsheet to Cytoscape (Supplementary: PPIP5K1_WT.sif)
4. We applied the BINGO application to assess molecular functions of proteins that interact with PPIP5K1^WT^.
5. We generated a protein association network using Agilent Literature text-mining application. We manually entered the list proteins that we identified in our screen into the application. Settings used: use of aliases accepted, and no context limit was set. As a result of text-mining, the application creates an association network where nodes are proteins and connections between nodes are created when proteins were associated with each other in literature (reported to interact, belong to the same pathway, found often in the same sentence). Protein-protein network generated by Agilent Literature search app (PPIP5K1_Agilent.sif) was merged with PPIPK1_WT.sif network. The resulting network was labeled in different colors depending on the most overrepresented molecular function of the proteins that are clustered together (Supplementary: PPIP5K1_VIN.sif)

**Movie M1** *Scratch assay of HeLa cells treated with control siRNA*. HeLa cells transfected with 25 nM non-targeting siRNAs (siCtrl) and pEGFP were grown to a monolayer and scratches were made with a micropipette tip. Wound closing was monitored for 15 hours post scratch as cells were placed in live-cell chamber under controlled conditions (37°C and 5% CO_2_). Real-time images were taken with an Operetta High Content Microscope (Perkin Elmer, Woodbridge, Ontario).

**Movie M2** *Scratch assay of HeLa cells treated with siPPIP5K1.* HeLa cells transfected with siRNA targeting PPIP5K1and EGFP empty vector were grown to a monolayer and scratches were made with a micropipette tip. Wound closing was monitored for 15 hours post scratch as cells were placed in live-cell chamber under controlled conditions (37°C and 5% CO_2_). Real-time images were taken with an Operetta High Content Microscope (Perkin Elmer Woodbridge, ON, Canada).

**Movie M3** *Scratch assay of HeLa cells treated siPPIP5K1 and co-transfected with PPIP5K1.* HeLa cells transfected with siRNA targeting PPIP5K1 and PPIP5K1 WT vector were grown to a monolayer and scratches were made with a micropipette tip. Wound closing was monitored for 15 hours post scratch as cells were placed in live-cell chamber under controlled conditions (37°C and 5% CO_2_). Real-time images were taken with an Operetta High Content Microscope (Perkin Elmer Woodbridge, ON, Canada). t

n
