# Supplementary figures and images for: Mass spectrometry analysis of PPIP5K1 interactions and data on cell motility of PPIP5K1-deficient cells
Source: Data Brief. 2016 Apr 2;7:1443–6. doi: 10.1016/j.dib.2016.03.035 (PMC5063796; doi:10.1016/j.dib.2016.03.035)

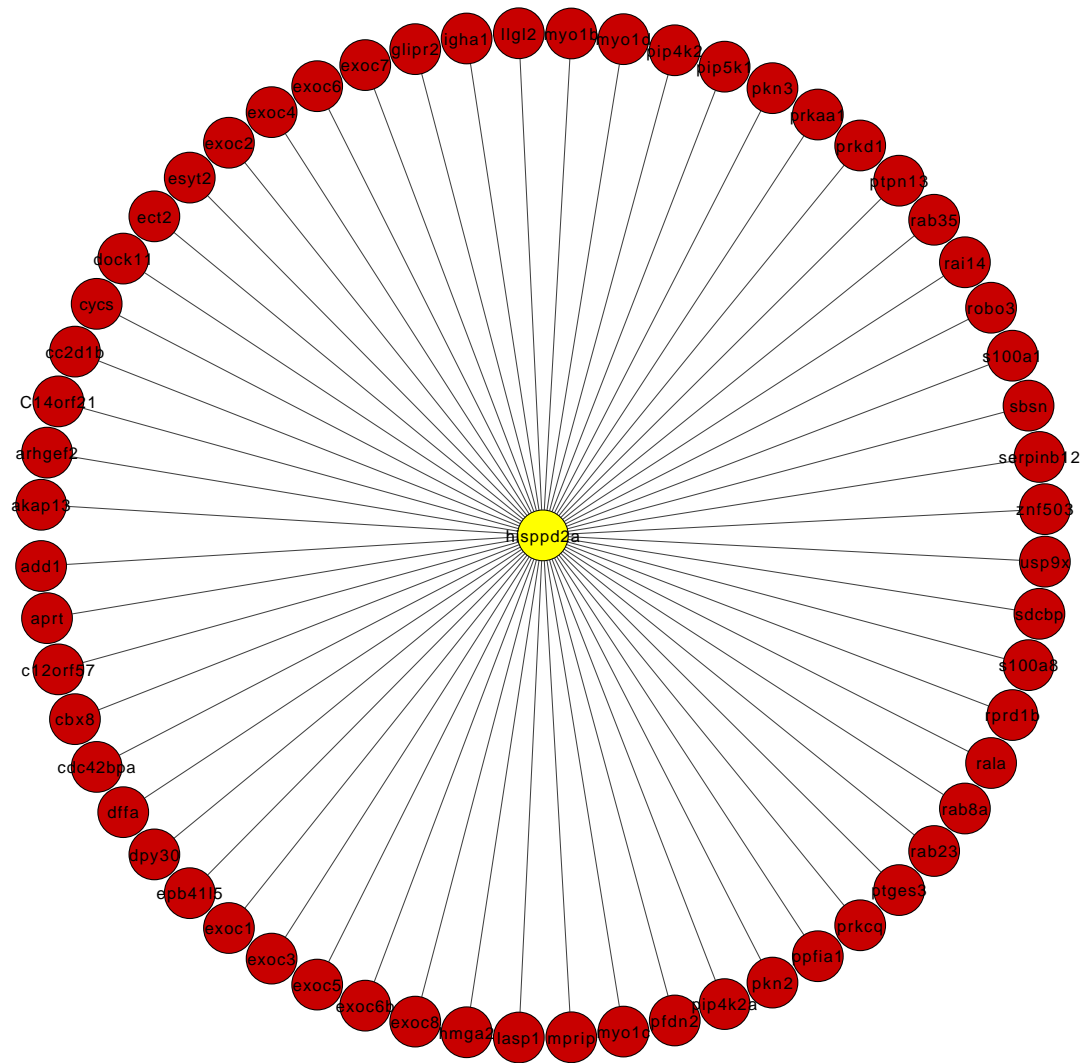

Supplement: Supplementary file 1 — Supplementary material [file mmc1.zip › Data in Brief_Proteomics/PPIP5K1_WT.pdf]
